# O Papel dos Marcadores Hemodinâmicos, Metabólicos e Biomarcadores na Previsão da Mortalidade após a Cirurgia de Revascularização do Miocárdio: Já Chegamos Lá?

**DOI:** 10.36660/abc.20240117

**Published:** 2024-04-09

**Authors:** Nelson Americo Hossne

**Affiliations:** 1 Universidade Federal de São Paulo Escola Paulista de Medicina Disciplina de Cirurgia Cardiovascular São Paulo SP Brasil Disciplina de Cirurgia Cardiovascular, Escola Paulista de Medicina, Universidade Federal de São Paulo, São Paulo, SP – Brasil

**Keywords:** Revascularização Miocárdica/cirurgia, Mortalidade, Biomarcadores/análise, Inflamação

A cirurgia de revascularização do miocárdio (CRM) é o procedimento cirúrgico cardíaco mais realizado no Brasil^1^ e em todo o mundo^2^ e tem sido um dos procedimentos mais estudados. Os aspectos pós-procedimento têm sido amplamente investigados e os escores de risco pré-operatório desenvolvidos. Vários marcadores e índices pós-operatórios, incluindo hemodinâmicos, metabólicos e biomarcadores, foram identificados e propostos, com o objetivo de prever com precisão os maus resultados dos pacientes. ^3 - 5^

Dr. Yamaguti et al.^6^ realizaram um estudo observacional prospectivo no qual foram analisados biomarcadores hemodinâmicos, metabólicos e de hipoperfusão tecidual em 183 pacientes (idades entre 35 e 83 anos) com disfunção ventricular esquerda (fração de ejeção entre 40% e 42,5%) submetidos à cirurgia de revascularização do miocárdio com circulação extracorpórea, conforme evolução clínica pós-operatória (complicada ou não complicada). Os pacientes com evolução clínica complicada (definida como morte dentro de 30 dias após a cirurgia ou mais de 4 dias de internação na UTI) eram mais velhos (66,3 vs 59,7; p = 0,002) e tinham um EuroSCORE mais alto (6 vs 3; p < 0,001). Os autores também constataram que o EuroSCORE, os níveis de lactato 6 horas após admissão na UTI, a diferença de pressão parcial de dióxido de carbono venoarterial (ΔPCO2) e o quociente respiratório estimado (eRQ = ΔPCO2/Ca-vO2) 12 horas após a admissão na UTI foram preditores independentes de evolução pós-operatória complicada, por análise de regressão logística multivariada.

Outros fatores contribuem de forma independente para piores resultados após a CRM. Os escores EuroSCORE II e STS têm bom poder de discriminação para prever mortalidade hospitalar de pacientes submetidos a CRM.^7^ Os níveis de proteína C reativa^4^ e lactato no pós-operatório^8^ estão diretamente correlacionados com maiores taxas de mortalidade hospitalar e complicações. Os níveis de troponina e CK-MB são fortes preditores de mortalidade em curto e longo prazo, independentemente do uso de circulação extracorpórea.^9^

Vários estudos e registros demonstraram que o tempo prolongado de pinçamento aórtico tem impacto prognóstico nesses pacientes,^10 , 11^ aumentando a mortalidade hospitalar e tardia. Além disso, as transfusões de sangue aumentam inadvertidamente os biomarcadores inflamatórios e de hipoperfusão, levando a um maior tempo de internação pós-operatória e maior morbimortalidade.^12 , 13^

Identificar parâmetros hemodinâmicos e metabólicos, bem como biomarcadores, muitos dos quais estão prontamente disponíveis e acessíveis em um ambiente de UTI, e compreender seu papel e correlação com os resultados pós-operatórios do paciente é de suma importância para melhorar o atendimento ao paciente, otimizando a perfusão tecidual e a extração de oxigênio.

No entanto, a resposta inflamatória após cirurgia cardíaca é heterogênea, mediada por uma rede complexa, e deve-se reconhecer o papel da imunidade inata na lesão de tecidos e órgãos após o procedimento. Uma revisão sistemática de ensaios clínicos randomizados sobre intervenções de proteção de órgãos visando a ativação do sistema imunológico inato não elucidou a incerteza quanto à eficácia desses tratamentos em cirurgia cardíaca.^14^

Nenhum biomarcador único como lesão tecidual relacionada especificamente a alterações inflamatórias intraoperatórias foi identificado,^15^ nem devemos visar um único caminho. A melhor abordagem hoje continua sendo a análise abrangente de diversos biomarcadores, juntamente com parâmetros hemodinâmicos e metabólicos.

## References

[1] Paez RP, Hossne NA, Santo JA, Berwanger O, Santos RH, Kalil RA (2019). Coronary artery by-pass surgery in Brazil: Analysis of the national reality through the by-pass registry. Braz J Cardiovasc Surg.

[2] Mack MJ, Squirs JJ, Lytle BW, DiMaio JM, Mohr FW (2021). Myocardial revascularization surgery: JACC historical breakthroughs in perspective. J Am Coll Cardiol.

[3] Shahian DM, Grover FL (2014). Biomarkers and risk models in cardiac surgery. Circulation.

[4] Heo RH, Wang MK, Meyre PB, Birchenough L, Park L, Vuong K (2023). Associations of inflammatory biomarkers with the risk of morbidity and mortality after cardiac surgery: a systematic review and meta-analysis. Can J Cardiol.

[5] Ramkumar N, Jacobs JP, Berman RB, Parker DM, MacKenzie TA, Likosky DS (2019). Cardiac biomarkers predict long-term survival after cardiac surgery. Ann Thorac Surg.

[6] Yamaguti T, Auler JOC, Dallan LAO, Galas FRBG, Cunha LGC, Piccioni MA (2024). Markers of Tissue Perfusion as Predictors of Adverse Outcomes in Patients with Left Ventricular Dysfunction Undergoing Coronary Artery Bypass Surgery. Arq Bras Cardiol.

[7] Gao F, Shan L, Wang C, Meng X, Chen J, Han L (2021). Predictive Ability of European Heart Surgery Risk Assessment System II (EuroSCORE II) and the Society of Thoracic Surgeons (STS) Score for in-Hospital and Medium-Term mortality of patients undergoing coronary artery by-pass grafting. Int J Gen Med.

[8] Hajjar LA, Almeida JP, Fukushima JT, Rhodes A, Vicent JL, Osawa EA (2013). High lactate levels are predictors of major complications after cardiac surgery. J Thorac Cardiovasc Surg.

[9] Petaja L, Salmenpera M, Pulkki K, Pettila V (2009). Biochemical injury markers and mortality after coronary artery by-pass grafting: a Systematic review. Ann Thorac Surg.

[10] Ruggieri VG, Bounader K, Verhoye JP, Onorati F, Rubino AS, Gatti G (2018). Prognostic impacto f prolonged cross-clamp time in coronary artery by-pass grafting. Heart Lung Circ.

[11] Doenst T, Borger MA, Weisel RD, Yau TM, Maganti M, Rao V (2008). Relation between aortic cross-clamp time and mortality - not as straightforward as expected. Eur J Cardio Thorac Surg.

[12] Santos AA, Sousa AG, Thomé HO, Machado RL, Piotto RF (2013). Impacto n eraly and late mortality after blood transfusion in coronary artery bypass graft surgery. Rev Bras Cir Cardiovasc.

[13] Murphy GJ, Reeves BC, Rogers CA, Rizvi SI, Culliford L, Angelini GD (2007). Increased mortality, postoperative morbidity, and cost after red blood cell transfusion in patients having cardiac surgery. Circulation.

[14] Abbasciano RG, Tomassini S, Roman MA, Rizzello A, Pathak S, Ramzi J (2023). Effects of interventions targeting the systemic inflammatory response to cardiac surgery on clinical outcomes in adults. Cochrane Database Sys Rev.

[15] Aljure OD, Fabbro II (2019). Cardiopulmonary bypass and inflammation: The Hidden Enemy. J Cardiotor Vasc Anest.

