# Supplementary material for: O Papel dos Marcadores Hemodinâmicos, Metabólicos e Biomarcadores na Previsão da Mortalidade após a Cirurgia de Revascularização do Miocárdio: Já Chegamos Lá?
Source: Arq Bras Cardiol. 2024 Apr 9;121(2):e20240117. [Article in Portuguese] doi: 10.36660/abc.20240117 (PMC11081087; doi:10.36660/abc.20240117)
Supplement: Translation

# Translation: The Role of Hemodynamic, Metabolic, and Biomarkers in Predicting Mortality after Coronary Artery Bypass Grafting: Are we there yet?

**Authors:** Nelson Americo Hossne

**Affiliations:** 1 São Paulo SP Brazil Disciplina de Cirurgia Cardiovascular, Escola Paulista de Medicina, Universidade Federal de São Paulo, São Paulo, SP – Brazil

**Keywords:** Myocardial Revascularization/surgery, Mortality, Biomarkers/analysis, Inflammation

Coronary artery bypass grafting (CABG) is the most common cardiac surgical procedure performed in Brazil^1^and worldwide^2^and has been one of the most studied procedures. Postprocedural aspects have been widely investigated and preoperative risk scores have been developed. Several postoperative markers and indexes, including hemodynamic, metabolic, and biomarkers, have been identified and proposed, aiming to accurately predict poor patient outcomes.^3 - 5^

Dr. Yamaguti et al.^6^conducted a prospective observational study in which hemodynamic, metabolic, and tissue hypoperfusion biomarkers were analyzed in 183 patients (ages between 35 and 83 years) with left ventricular dysfunction (ejection fraction between 40% and 42,5%) who underwent coronary artery bypass surgery with cardiopulmonary bypass, according to postoperative clinical course (complicated or uncomplicated). Patients with a complicated clinical course (defined as death within 30 days after surgery or more than 4 days of ICU stay) were older (66,3 vs 59,7; p=0.002) and had a higher EuroSCORE (6 vs 3; p<0.001). The authors also found that EuroSCORE, the lactate levels 6 hours after ICU admission, the venoarterial carbon dioxide partial pressure difference (ΔPCO2), and estimated respiratory quotient (eRQ = ΔPCO2/ Ca-vO2) 12 hours after ICU admission were independent predictors of complicated postoperative course, by multivariate logistic regression analysis.

Other factors independently contribute to worse outcomes after CABG. EuroSCORE II and STS scores have good discrimination power to predict in-hospital mortality of patients undergoing CABG.^7^Postoperative C-reactive protein^4^and lactate levels^8^are directly correlated to higher in-hospital mortality rates and complications. Troponin and CK-MB levels are stronger predictors of short and long-term mortality, regardless of the use of cardiopulmonary bypass.^9^

Several studies and registries demonstrated that a prolonged aortic cross-clamp time has a prognostic impact on these patients,^10 , 11^increasing in-hospital and late mortality. Moreover, blood transfusions inadvertently increase inflammatory and hypoperfusion biomarkers, leading to longer postoperative length of stay and higher morbimortality.^12 , 13^

Identifying hemodynamic and metabolic parameters, as well as biomarkers, most of which are readily available and accessible in a regular ICU setting, and understanding their role and correlation to patient postoperative outcomes is of paramount importance to improving patient care, by optimizing tissue perfusion and oxygen extraction.

Nevertheless, inflammatory response after cardiac surgery is intricate, mediated by a complex network, and one should acknowledge the role of innate immunity in tissue and organ injury following the procedure. A systematic review of RCTs of organ protection interventions targeting innate immune system activation did not resolve uncertainty as to the effectiveness of these treatments in cardiac surgery.^14^

No single biomarker as tissue injury related specifically to intraoperative inflammatory changes has been identified,^15^nor we should target a single pathway. The best approach nowadays remains the comprehensive analysis of several biomarkers, along with hemodynamic and metabolic parameters.
